# Foreword: Pathogens and immune responses of fish and reptiles

**DOI:** 10.1186/1297-9716-42-101

**Published:** 2011-09-21

**Authors:** Gael Kurath, Alexandra Adams

**Affiliations:** 1 USGS Western Fisheries Research Center, USA; 2 Institute of Aquaculture, University of Stirling, Scotland, UK

## Editorial

Fish and reptiles host a fascinating array of pathogens and diseases. Our knowledge of the host-pathogen interactions in these animals is driven by their importance in human food systems, zoological collections, medical products, research models, and healthy natural ecosystems. Although generally less extensively studied than mammalian and avian systems, the "lower vertebrates", which include fish, amphibians, and reptiles, have unique features of general veterinary interest. In the tree of life bony fishes are the earliest animals to have evolved adaptive immunity. Thus fish and reptiles have humoral and cellular immune systems as well as innate immunity, and they are important subjects for comparative immunology research. Lower vertebrates are also poikilothermic, or cold-blooded, so the function of the host immune response varies with environmental temperature, and their pathogens replicate with a range of temperature optima that are adapted to those of their specific hosts. Therefore the severity of disease is strongly impacted by temperature, and it is predicted that the disease ecology of these hosts may be particularly susceptible to changes due to large-scale effects of global warming. Research into fish and reptile diseases is conducted as field studies and laboratory experiments in living animals, in cultured cell lines, and at the molecular biology and in silico levels. For many diseases the research communities are small enough that diagnosticians, basic researchers, and policy makers all know each other and work together professionally to facilitate informed disease management. Due to the world-wide expansion of aquaculture, global transport of animals for the pet trade, and several significant disease emergence events in fish and reptiles, these are very active fields of study.

The virology papers in this special issue were inspired by keynote presentations at the 8th International Symposium on Viruses of Lower Vertebrates, held in Santiago de Compostela, Spain, in April of 2010. Together with the contributions on bacterial diseases, our intent is to present some of the most interesting and significant aspects of current research in fish and reptile disease and immunity. The co-editors appreciate the interest of *Veterinary Research *in presenting this special issue to the general veterinary scientific community, and gratefully acknowledge all the efforts of the authors whose work is presented here.

Sincerely,

Gael Kurath (USGS, Seattle, United States)

and

Alexandra Adams (University of Stirling, Scotland)

